# Supplementary material for: Anti-elite attitudes and support for independent candidates
Source: PLoS One. 2023 Oct 12;18(10):e0292098. doi: 10.1371/journal.pone.0292098 (PMC10569503; doi:10.1371/journal.pone.0292098)
Supplement: S1 File — (PDF) [file pone.0292098.s001.pdf]

Supplementary Appendix:
Anti-Elite Attitudes and Support for Independent Candidates

Pablo Argote* Giancarlo Visconti†

Contents

1 Appendix A: Defining Independents 3

2 Appendix B: Deviations from the Pre-Analysis Plan 4

3 Appendix C: Complete Results 8

4 Appendix D: Independents vs. Other Parties 10

5 Appendix E: Alternative Measure of Anti-Elite Attitudes 11

6 Appendix F: Conjoint Analysis with Controls 14

7 Appendix G: Inverse Probability Weighting 15

8 Appendix H: Marginal Means 18

9 Appendix I: Conjoint Diagnostics 20

10 Appendix J: Election Effect 22

11 Appendix K: External Validity 23

12 Appendix L: Text Analysis 28

*Post-doctoral researcher, Department of Political Science, University of Southern California, Los Angeles, California, United States; pablo.argotetironi@usc.edu.

†Assistant Professor, Department of Political Science, Pennsylvania State University; gvisconti@psu.edu

13 Appendix M: Comparative Study of Electoral Systems Data **29**

14 Appendix N: Days Until the Election **30**

1 Appendix A: Defining Independents

As discussed by Carreras (2012), there are conceptual difficulties associated with defining independents, outsiders, and populist politicians. Linz (1994, p.26) defined outsiders as "candidates not identified with or supported by any political party, sometimes without any governmental or even political experience, on the basis of a populist appeal often based on hostility to parties and politicians." Yet this definition does not clearly distinguish between independents, outsiders, and populist candidates. In this study, we treat them as different concepts.

We define *independent candidates* as individuals who run for office without the support of (or an affiliation with) a political party (Brancati, 2008; Rozas Bugueño et al., 2022). We can relax this definition for cases in which a recently created party supports a candidate (e.g., Alberto Fujimori supported by the ad hoc political party Cambio 90 in 1990). But if that candidate runs again with the same party's support, they should no longer be considered independent (e.g., Alberto Fujimori running for re-election in 1995). We understand *outsiders* as candidates who do not come from the political establishment and have not had a previous career in politics or public administration when the campaign starts (Carreras, 2012). Finally, we define *populism* as a political discourse that considers politics a moral struggle between the people and a "corrupt elite" (Rovira and Castiglioni, 2016).

Therefore, an independent candidate can be either an outsider, such as someone with no political experience (e.g., Alberto Fujimori in Peru in 1990), or an insider, such as someone who resigned from a political party to run without their support (e.g., Marco Enriquez-Ominami in Chile in 2009). Similarly, an independent candidate can use populist (e.g., Rodolfo Hernandez in Colombia in 2022) or ideological rhetoric (e.g., Jose Antonio Kast in Chile in 2017) to appeal to voters.

2 Appendix B: Deviations from the Pre-Analysis Plan

The pre-analysis plan (PAP) was registered with Open Science Framework on November 15, 2021, before the data collection concluded on November 20, 2021. We obtained access to the data on November 22, 2021.

In the PAP, we pre-registered the motivation, research question, hypotheses, methods, and analyses. As stated in the PAP, the main goal of the study is to investigate how non-institutional factors affect support for independent candidates. Below we discuss all the amendments to or deviations from the original pre-analysis plan.

Priming Experiment. The PAP included a priming experiment in which we exposed survey participants to scenarios that might affect their chances of voting for independents. We primed respondents with an anti-elite narrative before measuring their preferences for independent candidates using a conjoint experiment. The priming entailed explanations of the massive anti-elite protest that deeply shook the country's political structure in October 2019.

In the priming experiment, we randomly assigned subjects into one of four groups, exposing respondents to three interpretations of the 2019 protests, plus a pure control group. These narratives were deemed plausible causes of the social outbreak—see Araujo (2019) and Jiménez-Yañez (2020) for references—and they hold the elites responsible. The first narrative focuses on how the political elites were unable to incorporate individual social demands. The remaining two narratives allude to either the corruption among elites or the distance between economic elites and the rest of the population:

1. Crisis of Representation

   "One of the explanations used to understand the social outbreak is the **crisis of representation** of the political system in Chile. The political elite would have disconnected from the citizenry and would not have been able to incorporate important social demands that are very felt by the majority of Chileans."

2. Electoral Malfeasance

   "One of the explanations used to understand the social outbreak are the cases of **corruption and illegal financing of political campaigns** that exploded transversely in Chile in 2015. Both left- and right-wing politicians received deposits from companies such as Penta and SQM, through false receipts, to finance electoral campaigns for Congress."

3. Inequality

   "One of the explanations used to understand the social outbreak is the **socioeconomic inequality** in Chile. This problem is not restricted only to income or employment, but also includes issues such as education, health, dignity of treatment, and access to political and economic power."

However, a key problem with this design became apparent after pre-registration. These three political issues were already deeply settled in Chileans' minds after the 2019 social outburst. As a result, a null finding can be interpreted as (i) people not taking into account political issues such as the crisis of representation or (ii) people already having fixed anti-elite attitudes (i.e., ceiling effect). We report the results of the interaction between the priming and the conjoint experiment at the end of this section. We did not find evidence that the priming experiment affected people's electoral choices, but as mentioned above, we do not know if this is because the treatment is not relevant or if there is a ceiling effect in anti-establishment orientations.

Considering the limitations of the priming experiment, we decided to use a direct question to capture the anti-elite narratives that were present after the 2019 protests. We acknowledge that this new interaction was not pre-registered, but it is part of the original research question about how non-institutional variables help explain people's preferences. It also directly connects to the original priming experiment, which was designed to make people think about the anti-establishment sentiment that led to the 2019 social outburst.

The new measure is not affected by a ceiling effect since it does not capture *variation* in anti-elite attitudes but rather the *level* of this sentiment. However, this approach has shortcomings, namely the fact that anti-establishment attitudes are not randomly assigned. People with and without this sentiment can differ in multiple characteristics, which could impact the outcome of interest (i.e., voting for independents). To address some aspects of this concern, we use inverse probability weighting (IPW) to generate groups of anti-elite and non-anti-elite respondents with similar distributions of observed characteristics. We focus on key characteristics used in the literature to understand how Chileans vote, such as their socioeconomic status, education, gender, region, age, ideology, and previous electoral choices (Altman, 2004; López, 2004; Morales, 2010; Visconti, 2021).

Hypothesis and Analysis. We replaced the priming experiments with individuals' anti-elite attitudes in the hypotheses and the analysis. Now, the main results are based on an interaction between anti-establishment attitudes (rather than the priming experiment) and the conjoint experiment. The results of the whole conjoint experiment separated by priming can be found in Figure B1; Figure B2 displays the interaction between the vote for independents and each priming.

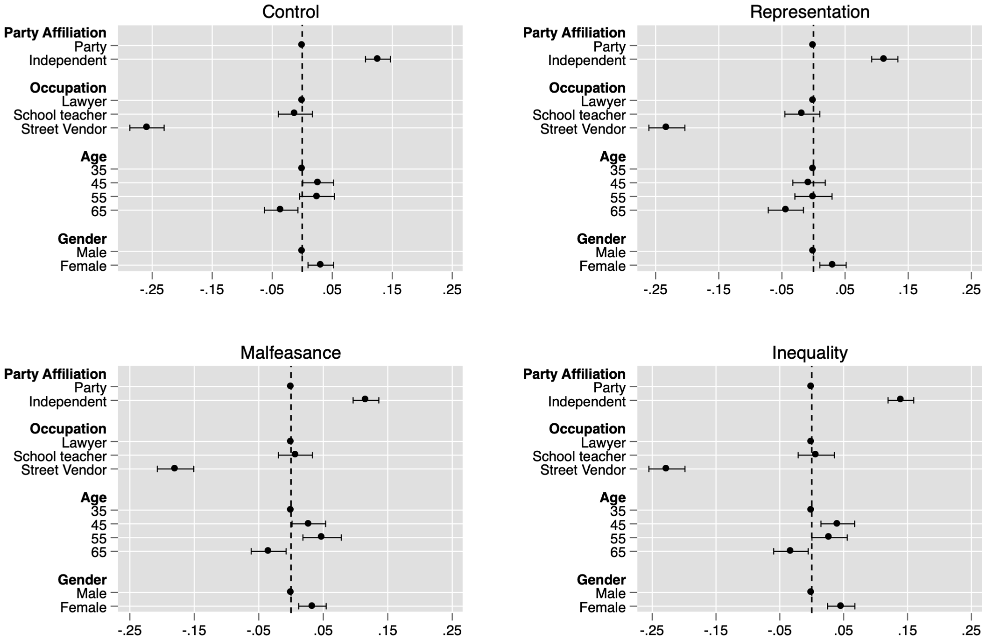

Figure B1: Conjoint Experiment Results: Per Priming Experiment
The outcome is the preference for a given candidate. Circles represent point estimates, and lines 95% confidence intervals. Standard errors are clustered at the respondent level

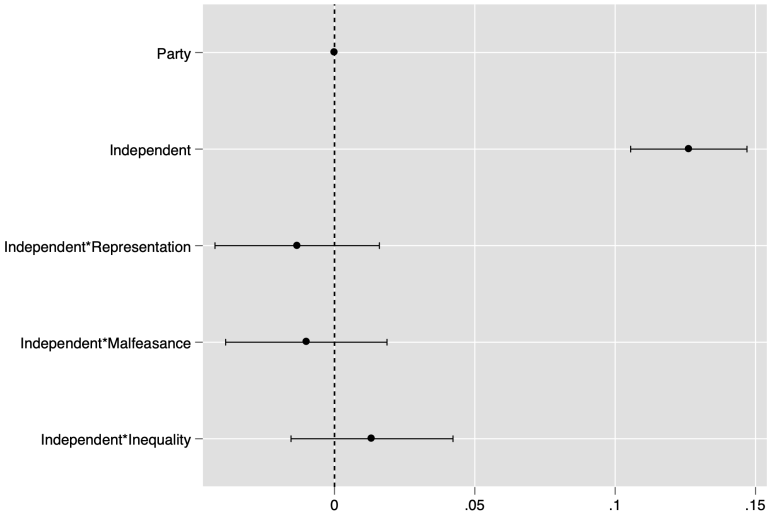

Figure B2: Conjoint Experiment Results: Interaction Priming*Vote for Independents
The outcome is the preference for a given candidate. Circles represent point estimates, and lines 95% confidence intervals. Standard errors are clustered at the respondent level

3 Appendix C: Complete Results

In this appendix, we present the complete results of the conjoint experiment. Figure C1 shows a coefficient plot with each attribute, whereas Figure C2 also displays the interaction term between the attributes and non-elite attitudes. Finally, Table C1 reports the numerical values of each coefficient for both the interacted and non-interacted models.

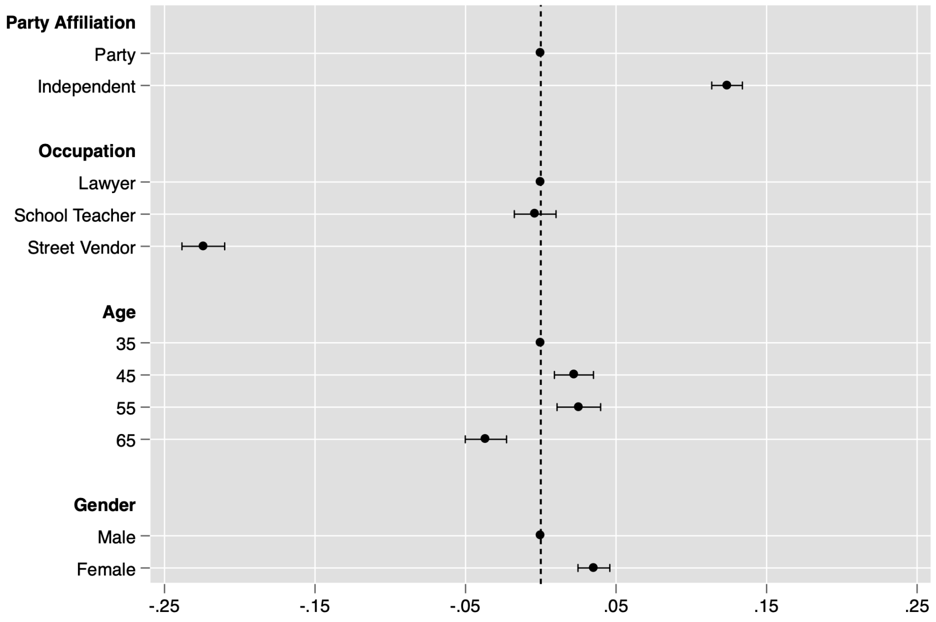

Figure C1: Conjoint Experiment Results: All Attributes
The outcome is the preference for a given candidate. Circles represent point estimates, and lines 95% confidence intervals. Standard errors are clustered at the respondent level

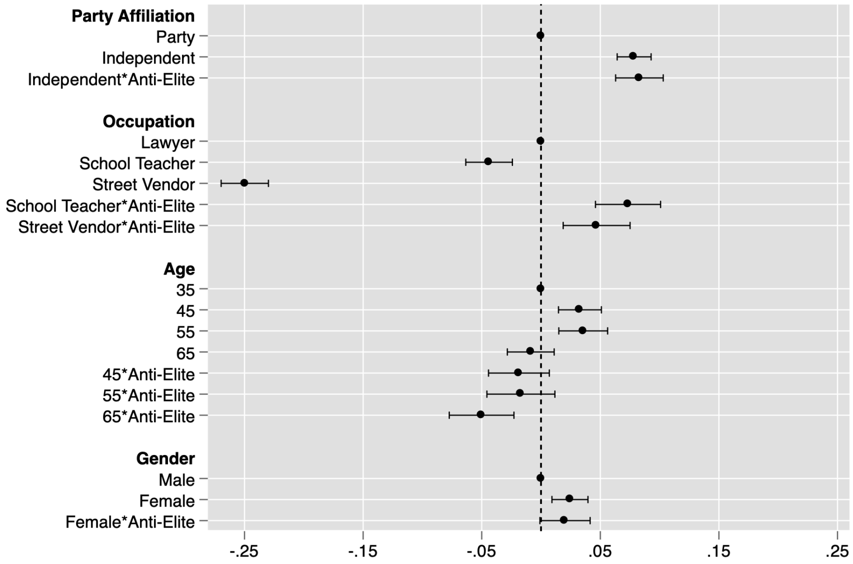

Figure C2: Conjoint Experiment Results: All Attributes (Interaction with Anti-Elite attitudes)
The outcome is the preference for a given candidate. Circles represent point estimates, and lines 95% confidence intervals. Standard errors are clustered at the respondent level

	(1) Non-Interacted	(2) Interacted
Independent	0.124***	0.073***
	(0.005)	(0.007)
School Teacher	-0.004	-0.004
	(0.007)	(0.007)
Street Vendor	-0.224***	-0.233***
	(0.007)	(0.009)
Age = 45	0.022***	0.022**
	(0.007)	(0.009)
Age = 55	0.025***	0.024**
	(0.007)	(0.010)
Age = 65	-0.036***	-0.020**
	(0.007)	(0.010)
Female	0.035***	0.020***
	(0.005)	(0.007)
Anti-Elite*Independent		0.093***
		(0.010)
Anti-Elite*Street Vendor		0.015
		(0.011)
Anti-Elite*45		-0.000
		(0.012)
Anti-Elite*55		0.003
		(0.013)
Anti-Elite*65		-0.030**
		(0.012)
Anti-Elite*Female		0.028***
		(0.010)
R^2	0.0720	0.0787
Obs.	39650	39650

Table C1: Conjoint Experiment Results: All Attributes (Interaction with Anti-Elite Attitudes)
The outcome is the preference for a given candidate. The omitted attributes of the conjoint are party member, lawyer, 35 years of age, and male. Standard errors are clustered at the respondent level

4 Appendix D: Independents vs. Other Parties

In this section, we present the preference for independents vs. parties of the left, center-left, center-right, and right (Figure D1). We use the independent category as the baseline. The figure illustrates that respondents preferred independents to all the remaining political parties, although the gap is more pronounced for center-right parties.

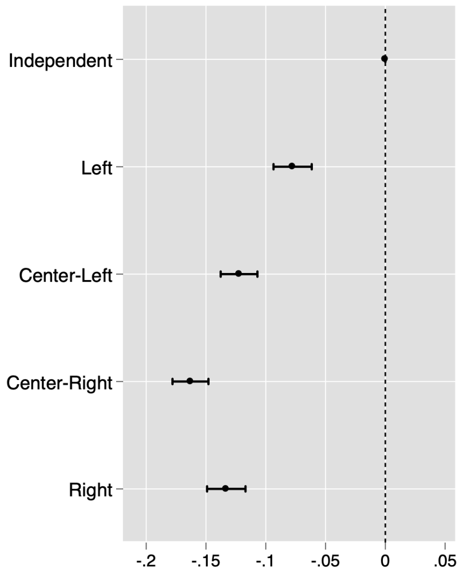

Figure D1: Preference for Independents Compared to Political Parties
The outcome is the preference for a given candidate. The other conjoint attributes are omitted. Independent is the baseline. The dots represent point estimates, and lines 95% confidence intervals. Standard errors are clustered at the respondent level

5 Appendix E: Alternative Measure of Anti-Elite Attitudes

In this section, we present robustness checks of our main results. To determine whether our results are an artifact of how we measure anti-elite attitudes, we present the coefficient of preferences for independents for respondents with and without anti-elite attitudes, using two alternative ways to measure such attitudes (see Figures E1 and E2). The figure notes describe the measures.

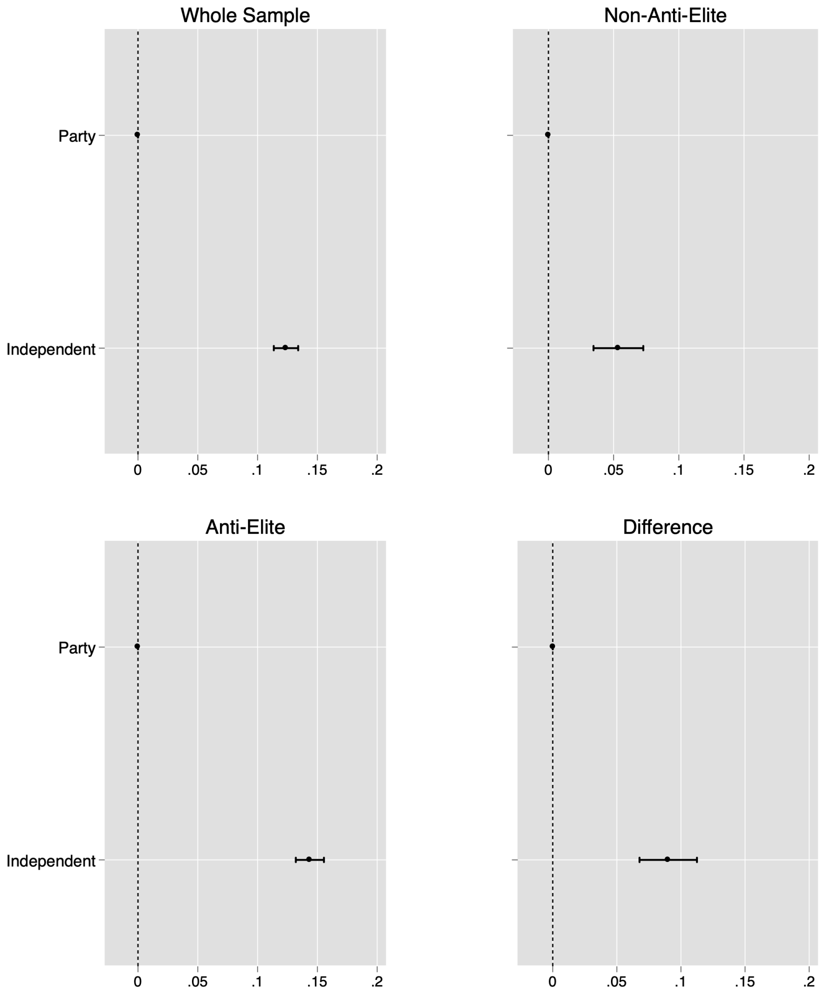

Figure E1: Conjoint Experiment Results: First Alternative Measure of Anti-Elite Attitudes
The outcome is the preference for a given candidate. The other conjoint attributes are omitted. We used the following question to measure anti-elite attitudes: "Below you will read two statements; which one is closer to your ideas? A) legislators should follow the will of the people when making laws B) legislators should follow their own knowledge and opinions when making laws." Respondents choosing alternative A) were considered populists. The dots represent point estimates, and lines 95% confidence intervals. Standard errors are clustered at the respondent level

Note that the 77% of respondents hold anti-elite attitudes according to our first alternative

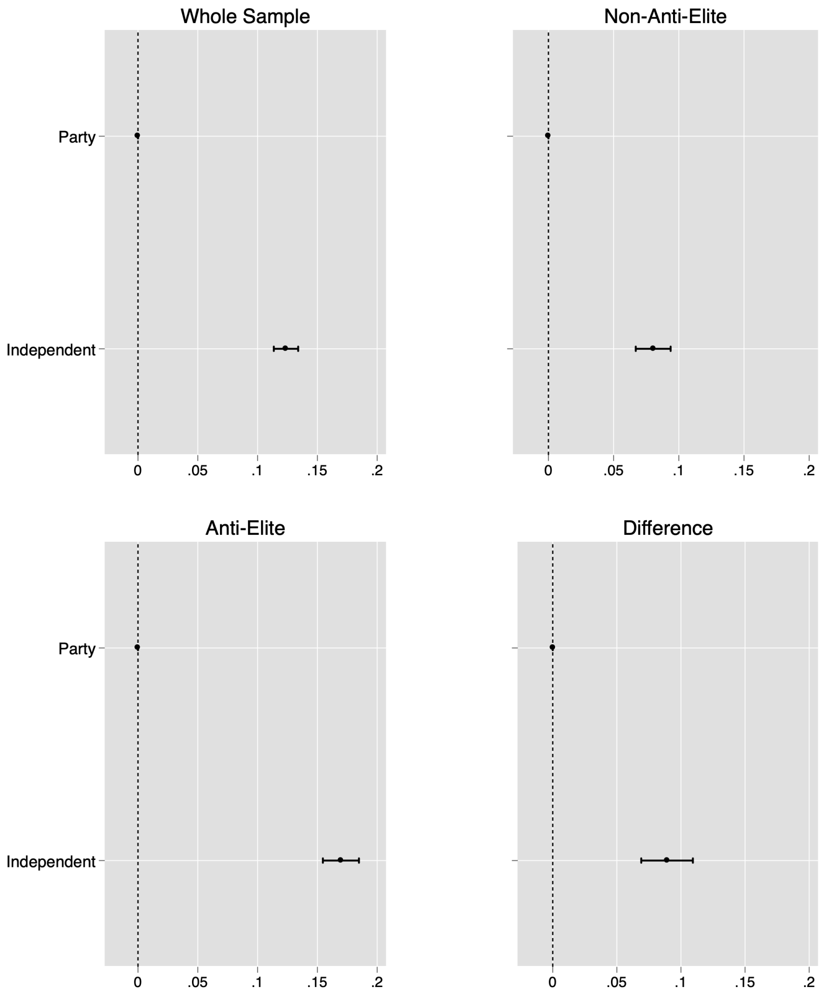

Figure E2: Conjoint Experiment Results: Second Alternative Measure of Anti-Elite Attitudes
The outcome is the preference for a given candidate. The other conjoint attributes are omitted. We used two questions to measure anti-elite attitudes: 1) "Below, you will read two statements; which one is closer to your ideas? A) legislators should follow the will of the people when making laws B) legislators should follow their own knowledge and opinions when making laws." 2) "Below, you will read two statements; which one is closer to your ideas? A) The main division in society is between the people and the elite. B) The main division in society is between the left and the right." Respondents who chose alternative A) in both questions were defined as anti-elite. The dots represent point estimates, and lines 95% confidence intervals. Standard errors are clustered at the respondent level

measure —the one about whether legislators should follow the will of the people or their own knowledge— which is very high. We also included a stricter definition of anti-elite attitudes by combining the two questions we have used to capture these orientations (see Figure E2). According to this latter measure, 48% of respondents hold anti-elite attitudes.

Finally, the correlation between the measure used in the paper and the first alternative measure is 0.28, which is not particularly high, likely because of the high percentage of people who believe that legislators should follow the will of the people instead of their own knowledge. Given that the later variable does not have too much variation, we decided to focus, in the manuscript, on the former (i.e., what is the main division in society). In any case, the conclusions of the study are not conditional on how we measure anti-elite attitudes.

6 Appendix F: Conjoint Analysis with Controls

In this appendix, we present our main results using a set of demographic control variables. The results are practically identical to those presented in the main manuscript (see Figure F1).

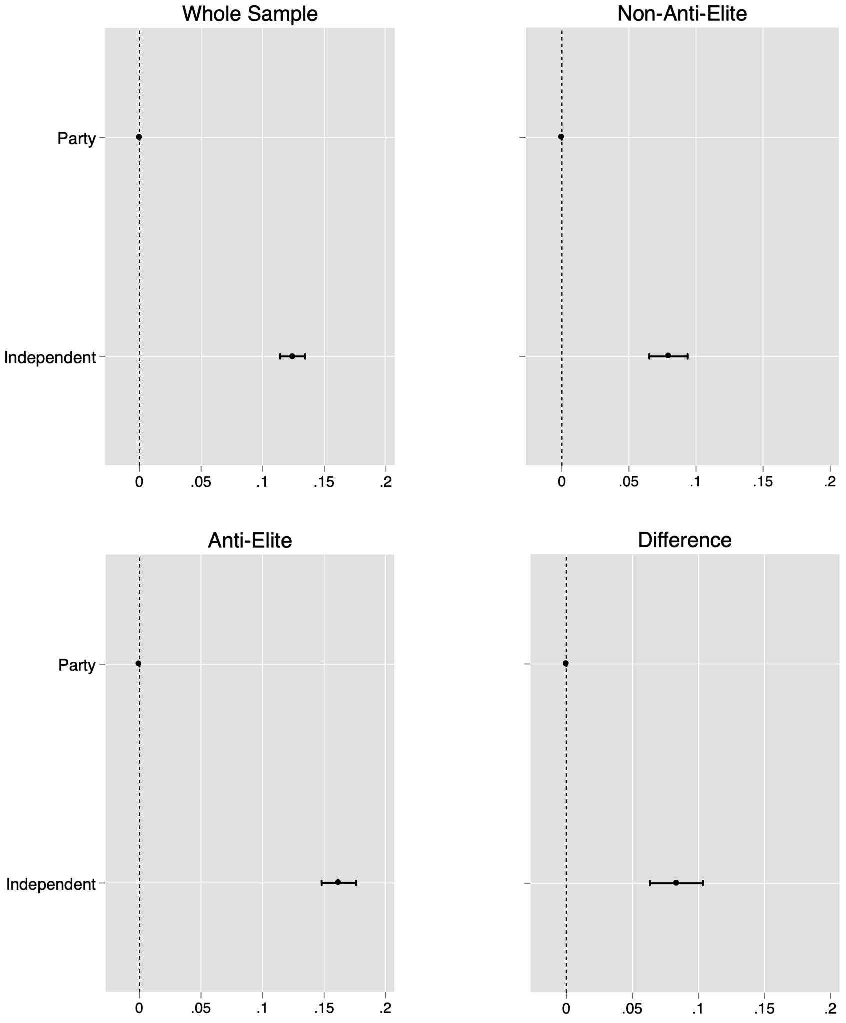

Figure F1: Conjoint Experiment Results: Using Control Variables
The outcome is the preference for a given candidate. The other conjoint attributes are omitted (see Appendix A for the complete non-interacted and interacted results). Control variables are region, age, gender, education, socioeconomic status, duration (in minutes), and device (desktop computer or cell phone). The dots represent point estimates, and lines 95% confidence intervals. Standard errors are clustered at the respondent level

7 Appendix G: Inverse Probability Weighting

To rule out the possibility that anti-elite attitudes would capture either a demographic or political covariate, we estimated our main results, weighting each observation by the inverse probability of having anti-elite attitudes, conditional on demographic and political covariates (IPW). First, in Table G1, we show that respondents with anti-elite and non-anti-elite attitudes differ regarding these observable characteristics. For instance, people with anti-elite attitudes are, on average, younger.

Variable	(1) Non-Anti-Elite	(2) Anti-Elite	(3) Difference
SES (Ordinal)	5.125	5.150	0.025*
	(1.429)	(1.342)	(0.014)
Education (Ordinal)	5.857	5.988	0.131***
	(1.779)	(1.751)	(0.018)
Female	0.508	0.501	-0.007
	(0.500)	(0.500)	(0.005)
Santiago	0.398	0.404	0.007
	(0.489)	(0.491)	(0.005)
Age	44.456	41.527	-2.929***
	(16.981)	(16.228)	(0.167)
Duration (Minutes)	21.012	20.690	-0.322***
	(11.271)	(11.410)	(0.114)
Desktop Computer	0.745	0.735	-0.010**
	(0.436)	(0.441)	(0.004)
Left	0.111	0.317	0.206***
	(0.314)	(0.465)	(0.004)
Right	0.326	0.114	-0.212***
	(0.469)	(0.318)	(0.004)
Centrist	0.259	0.266	0.007*
	(0.438)	(0.442)	(0.004)
No ideology	0.304	0.303	-0.001
	(0.460)	(0.460)	(0.005)
Voted 2017	0.661	0.705	0.044***
	(0.473)	(0.456)	(0.005)
Observations	18,180	21,470	39,650

Table G1: Balance Table: Demographic and Survey-related Variables by Anti-Elite Attitudes (Before Inverse Probability Weighting)

However, after applying the weights, both groups are practically identical in these covariates (see Table G2). Moreover, Figure G1 shows that the paper's main results are very similar to those reported in the manuscript after applying IPW.

Variable	(1)<br>Non-Anti-Elite	(2)<br>Anti-Elite	(3)<br>Difference
SES (Ordinal)	5.161	5.146	-0.015
	(1.375)	(1.388)	(0.014)
Education (Ordinal)	5.911	5.923	0.012
	(1.766)	(1.769)	(0.018)
Female	0.508	0.506	-0.002
	(0.500)	(0.500)	(0.005)
Santiago	0.404	0.403	-0.001
	(0.491)	(0.491)	(0.005)
Age	42.639	42.662	0.023
	(16.630)	(16.629)	(0.167)
Duration (Minutes)	20.884	20.825	-0.059
	(11.014)	(11.248)	(0.112)
Desktop Computer	0.747	0.743	-0.003
	(0.435)	(0.437)	(0.004)
Left	0.220	0.222	0.002
	(0.414)	(0.416)	(0.004)
Right	0.211	0.210	-0.001
	(0.408)	(0.407)	(0.004)
Centrist	0.265	0.265	-0.001
	(0.442)	(0.441)	(0.004)
No ideology	0.303	0.303	-0.000
	(0.460)	(0.460)	(0.005)
Voted 2017	0.674	0.676	0.002
	(0.469)	(0.468)	(0.005)
Observations	18,150	21,460	39,610

Table G2: Balance Table: Demographic and Survey-related Variables by Anti-Elite Attitudes (After Inverse Probability Weighting)

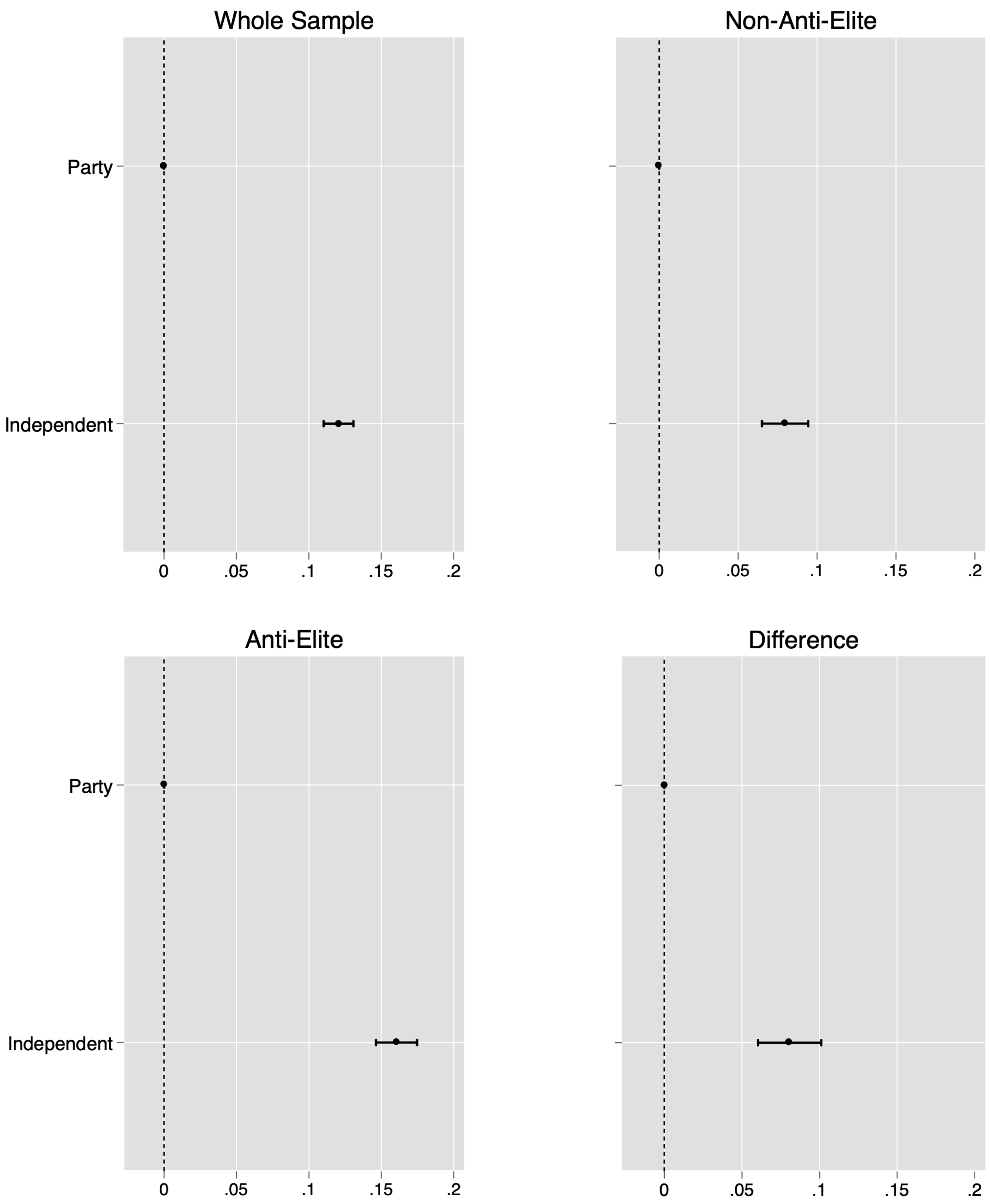

Figure G1: Conjoint Experiment Results: Inverse Probability Weighting
The outcome is the preference for a given candidate. The other conjoint attributes are omitted. The inverse probability weights were obtained by calculating the predicted values of a probit model regressing populist attitudes on region, age, gender, education, socioeconomic status, duration (in minutes), and device (desktop computer or cell phone). The dots represent point estimates, and lines 95% confidence intervals. Standard errors are clustered at the respondent level

8 Appendix H: Marginal Means

In this section, we present the results using marginal means instead of the AMCE. Since Chile had voluntary voting in 2021, our conjoint allowed respondents to refuse to vote for any candidate (i.e., outcome = 0 for both profiles). As a result, the benchmark for the marginal means is lower than 50%. In the left panel of Figure H2, we display the marginal mean coefficient for the whole sample, while the right panel separates the sample by anti-elite attitudes. The figure confirms that respondents with anti-elite attitudes are the driving force behind the preference for independents.

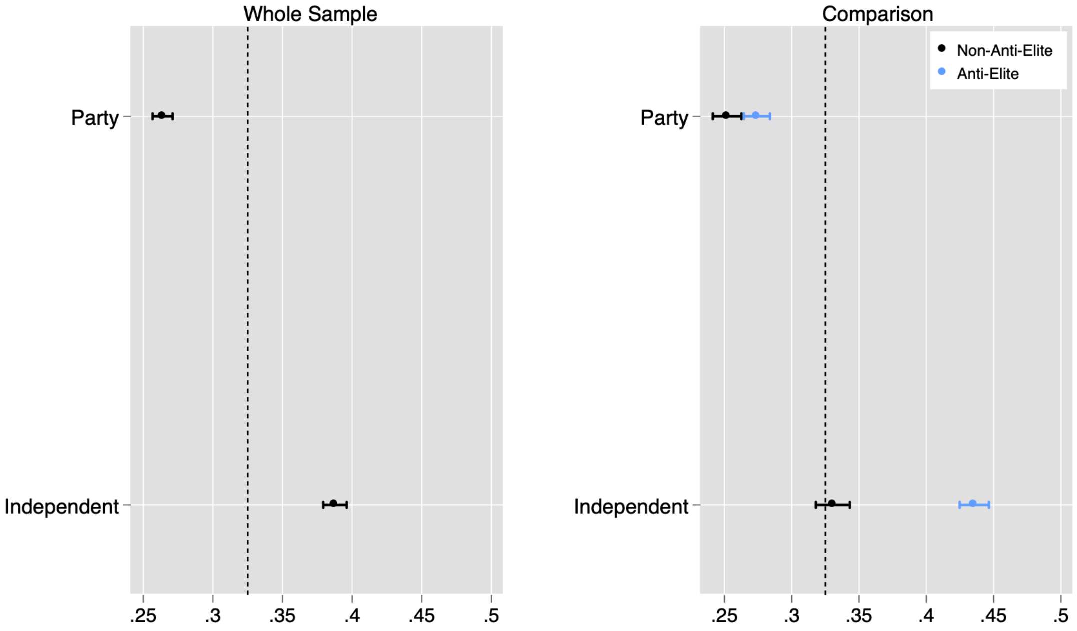

Figure H1: Preference for Independents by Populist Beliefs (Marginal Means)
The outcome is the preference for a given candidate. The other conjoint attributes are omitted (see Appendix C for the complete non-interacted and interacted results). Control variables are region, age, gender, education, socioeconomic status, duration (in minutes), and device (desktop computer or cell phone). The dots represent point estimates, and lines 95% confidence intervals. Standard errors are clustered at the respondent level

As these are marginal means—rather than regression coefficients—it is important to provide additional elements to interpret the point estimates. In the right panel, when evaluating the results for anti-elite respondents (blue coefficients), we see that the expected value of independent candidates is approximately 0.44, almost 18 percentage points higher than the expected value of party members. By contrast, among respondents without anti-elite attitudes (black coefficients),

the difference is much lower—although it still exists. People without anti-elite attitudes are actually indifferent between choosing an independent and someone at random. It is thus clear that respondents with anti-elite beliefs are driving the preference for independents.

Finally, we include the marginal means when using people who reported a preference in the conjoint experiment. Therefore, in this analysis, we exclude the non-voters. Again, results are consistent with previous findings, with anti-elite voters being more likely to prefer independent candidates than non-anti-elite voters. However, the difference becomes smaller, which is not surprising, considering that non-voters are more likely to be anti-elite. Furthermore, in 2023, Chile adopted mandatory voting; consequently, this group of anti-elite citizens will now be forced to participate in the elections making independent candidates more likely to be elected.

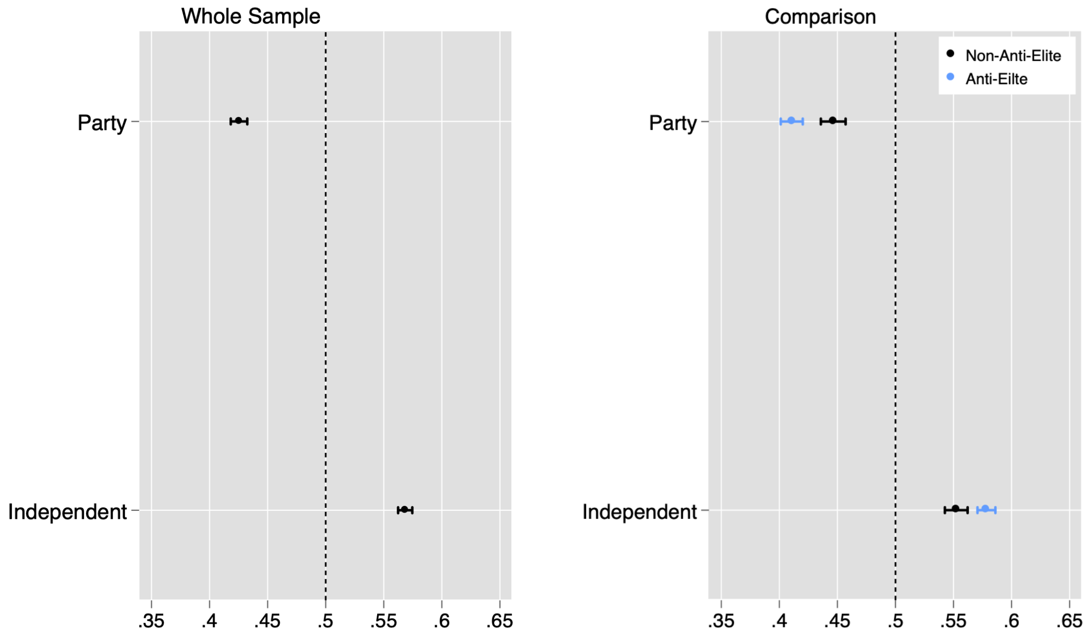

Figure H2: Preference for Independents by Populist Beliefs (Marginal Means)
The outcome is the preference for a given candidate. The other conjoint attributes are omitted (see Appendix C for the complete non-interacted and interacted results). Control variables are region, age, gender, education, socioeconomic status, duration (in minutes), and device (desktop computer or cell phone). The dots represent point estimates, and lines 95% confidence intervals. Standard errors are clustered at the respondent level. Non-voters are excluded from the analysis

9 Appendix I: Conjoint Diagnostics

In this appendix, we present the conjoint diagnostics. Table I1 displays the balance of each conjoint attribute against a set of demographic variables. We regressed the variables displayed in the columns on all the conjoint attributes. As expected, there is covariate balance when using these demographics as outcomes.

	(1)<br>Duration (Minutes)	(2)<br>Female	(3)<br>Age	(4)<br>SES (Ordinal)
Independent	-0.075	-0.004	-0.095	0.012
	(0.103)	(0.005)	(0.153)	(0.013)
School teacher	-0.008	-0.002	0.028	0.003
	(0.099)	(0.004)	(0.145)	(0.012)
Street Vendor	-0.016	0.000	0.084	-0.014
	(0.109)	(0.005)	(0.160)	(0.013)
Age = 45	0.069	-0.001	-0.107	-0.015
	(0.089)	(0.004)	(0.129)	(0.011)
Age = 55	-0.052	-0.001	0.050	-0.006
	(0.111)	(0.005)	(0.159)	(0.013)
Age = 65	0.147	-0.003	0.047	-0.025
	(0.128)	(0.006)	(0.184)	(0.016)
Female	-0.009	0.000	0.033	0.001
	(0.017)	(0.001)	(0.026)	(0.002)
Obs.	39650	39650	39650	39650

Table I1: Balance Table Key Covariates

We also estimate an additional conjoint diagnostic: whether appearing (1) on the left or right side of the screen (i.e., order effect) or (2) in the first, second, third, or fourth pair of the conjoint (i.e., wave effect) affected respondents' preferences for independent candidates. We find no systematic evidence of an order or wave effect; respondents prefer independents when they appear on either side of the screen and in every wave. There are some minor differences in the waves (wave 2), but they do not substantively alter the main results.

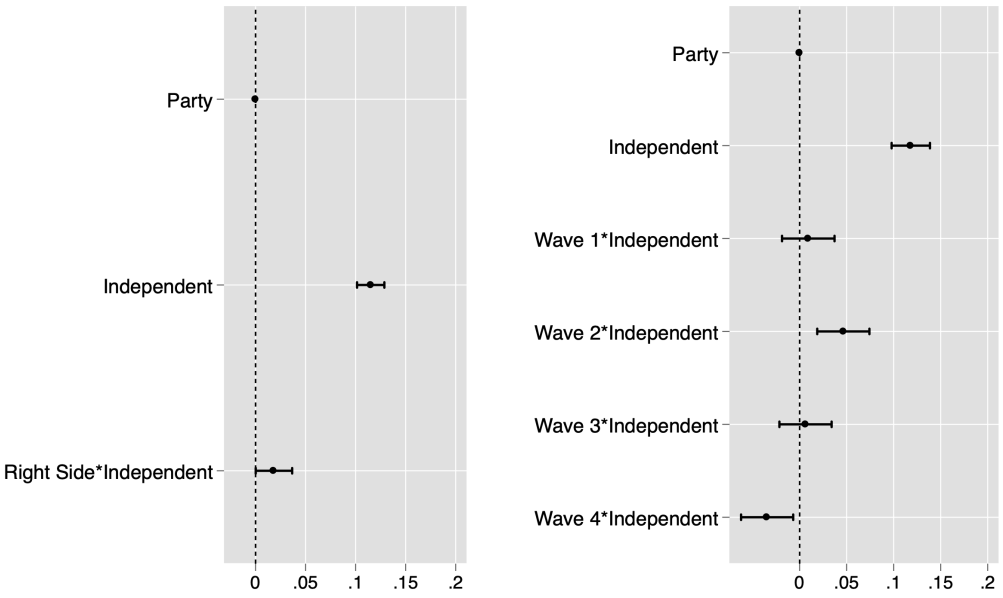

Figure I1: Conjoint Diagnostics: Order and Wave Effect

The outcome is the preference for a given candidate. The dots represent point estimates, and lines 95% confidence intervals. The omitted category in the left panel figure is the left side; in the right panel, the omitted category is the fifth wave. Standard errors are clustered at the respondent level

10 Appendix J: Election Effect

As a robustness check, we evaluate whether Chile's real-life campaign affected respondents' choices of hypothetical candidates in the conjoint experiment. We tested the potential impact of the number of days remaining until the 2021 presidential election and found that proximity to the election does not affect the preference for independents (Figure I2). As a note to the readers, the confidence intervals for the interaction are so small that they are hard to see in the figure.

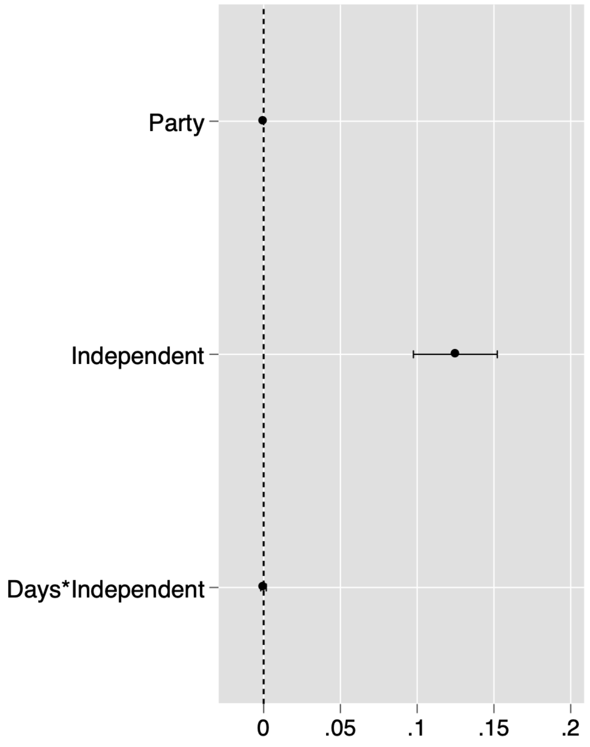

Figure I2: Conjoint Experiment Results: Interaction Independent*Days to Election
The outcome is the preference for a given candidate. The circle represents the point estimate, and the line the 95% confidence interval. Standard errors are clustered at the respondent level

11 Appendix K: External Validity

In this section, we address potential external validity issues with the Netquest sample. Table L1 indicates that the sample is generally aligned with the Chilean population in age and gender, although it under-represents some categories of education (less than high school and technical). Thus, we adjusted our result by post-stratification weights based on the counts of the combination of region, gender, education, and age. We also created a weight using the "raking" approach, which uses the marginal distributions of these variables instead of the per-cell count. The following results (see Figures K1, K2, K4, K5, K6) present the paper's main results, adjusted by post-stratification and rake weights. In general, the results are somewhat magnified, meaning that the preference for independent candidates among populists is greater after weighing the sample. Most likely, the effects increased because we assigned a higher weight to less educated respondents.

Table K1: External Validity Netquest Sample

	% 2017 Census	% Netquest
18-24	14.3	17.4
25-34	20.8	20.7
35-44	18.1	18.2
45-54	17.6	17.2
55-64	14.2	13.0
65-74	8.8	10.3
75 or more	6.3	3.3
Female	51.1	50.4
Male	48.9	49.6
Less than High School	24	5.4
High School	45	48.2
Technical	9.3	22.5
College or graduate	21.7	23.9

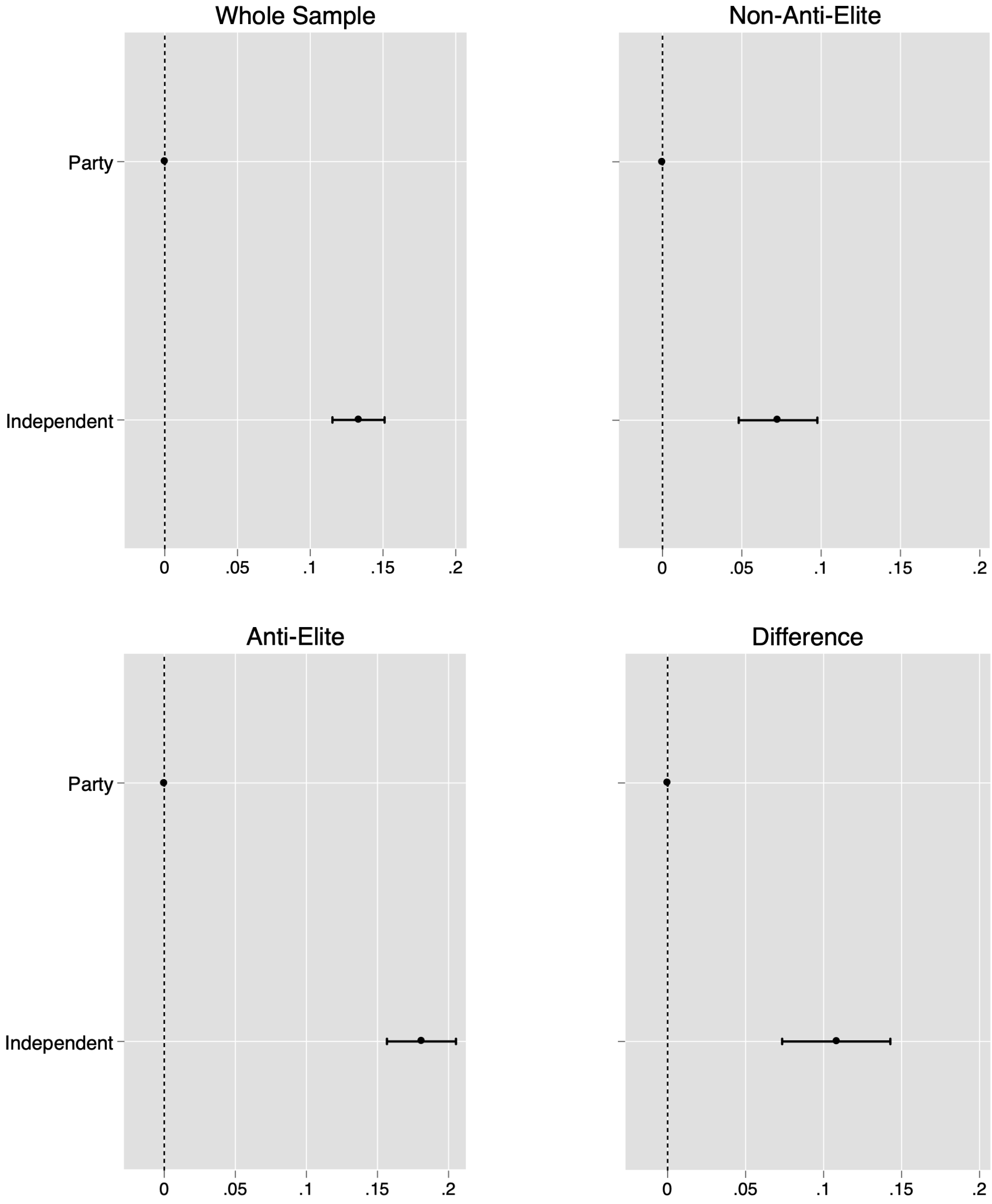

Figure K1: Preference for Independents by Populist Beliefs (Census-based Cell Weights)
The outcome is the preference for a given candidate. Circles represent point estimates, and lines 95% confidence intervals. Standard errors are clustered at the respondent level

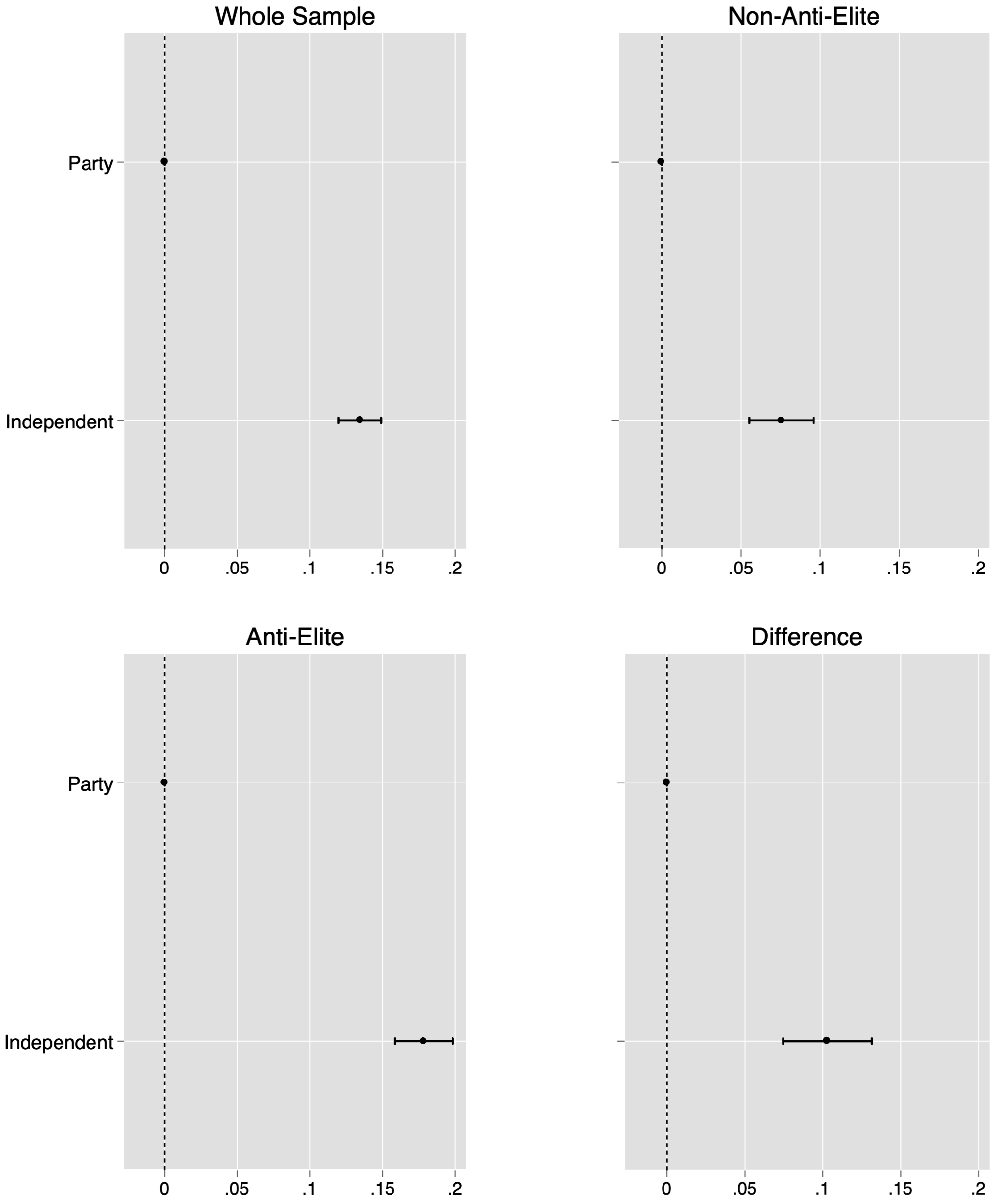

Figure K2: Preference for Independents by Populist Beliefs (Census-based Rake Weights)
The outcome is the preference for a given candidate. Circles represent point estimates, and lines 95% confidence intervals. Standard errors are clustered at the respondent level

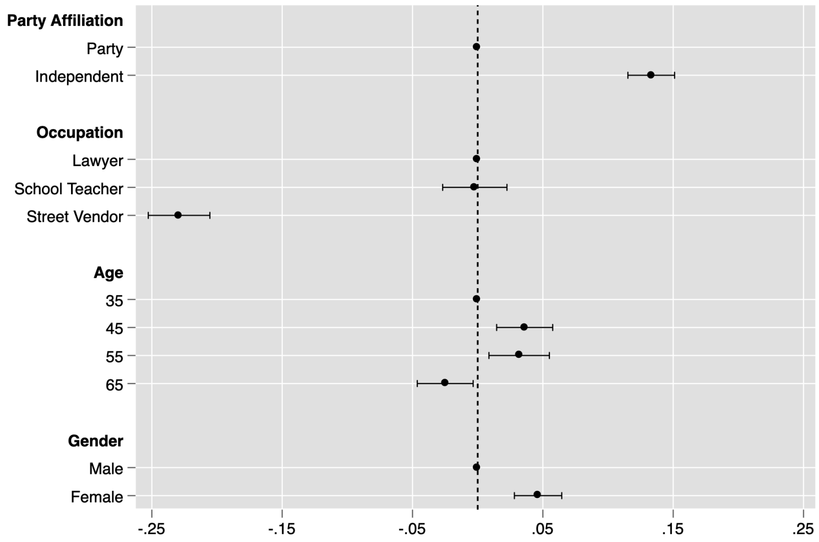

Figure K3: Conjoint Experiment Results: All Attributes (Census-Based Cell Weights)
The outcome is the preference for a given candidate. Circles represent point estimates, and lines 95% confidence intervals. Standard errors are clustered at the respondent level

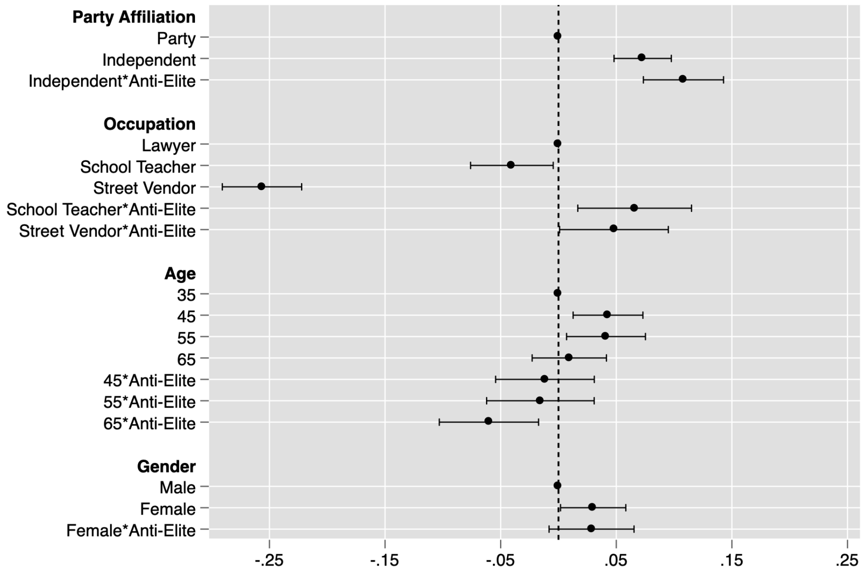

Figure K4: Conjoint Experiment Results: All Attributes (Interacted by Populist Beliefs, Census-Based Cell Weights)
The outcome is the preference for a given candidate. Circles represent point estimates, and lines 95% confidence intervals. Standard errors are clustered at the respondent level

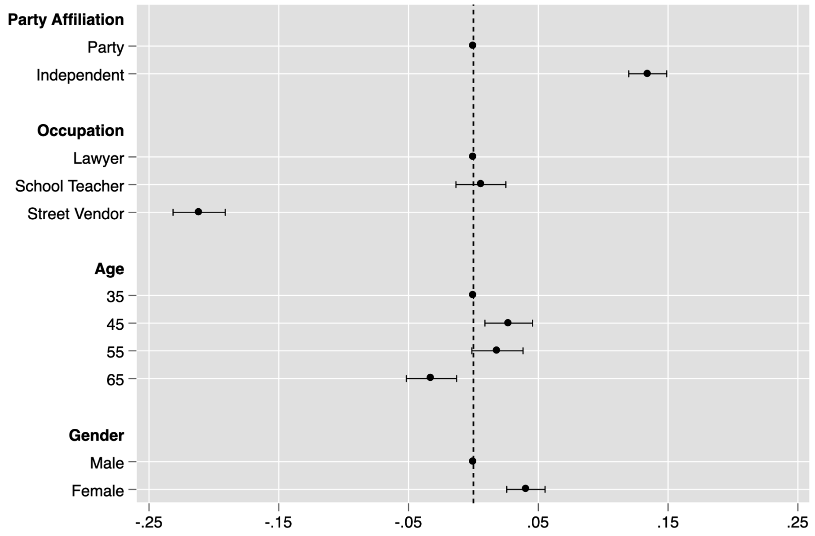

Figure K5: Conjoint Experiment Results: All Attributes (Census-Based Rake Weights)
The outcome is the preference for a given candidate. Circles represent point estimates, and lines 95% confidence intervals. Standard errors are clustered at the respondent level

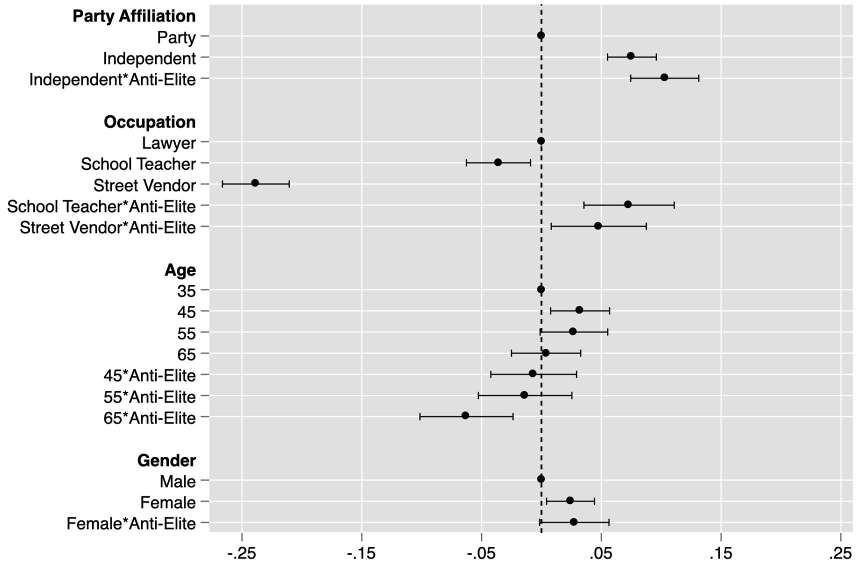

Figure K6: Conjoint Experiment Results: All Attributes (Interacted by Populist Beliefs, Census-Based Rake Weights)
The outcome is the preference for a given candidate. Circles represent point estimates, and lines 95% confidence intervals. Standard errors are clustered at the respondent level

12 Appendix L: Text Analysis

In this section, we provide a basic descriptive analysis of two open-ended questions: we asked respondents words associated with the terms "Party Members" and "Independent Politicians." We first provide two-word clouds in Spanish in Figures L1 and L2.

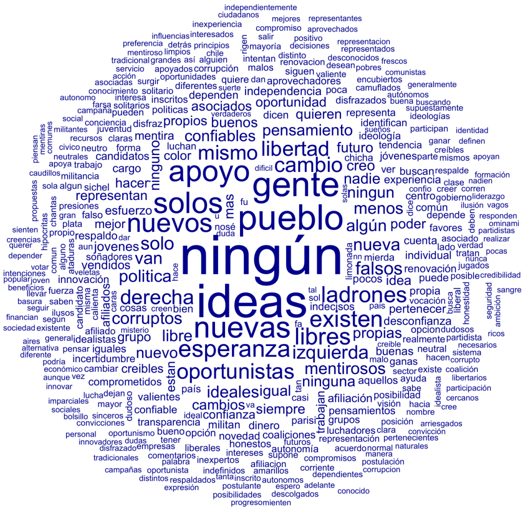

Figure L1: Word Cloud: Independents

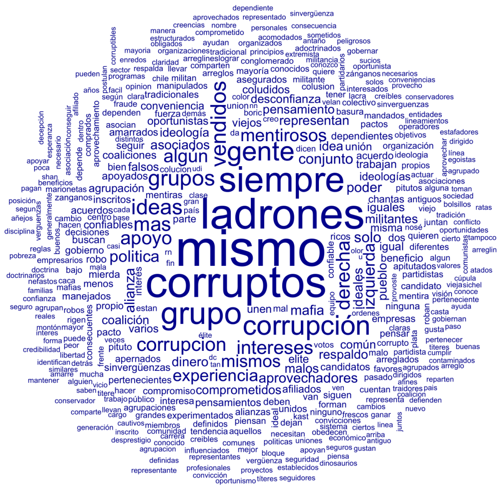

Figure L2: Word Cloud: Party Members

13 Appendix M: Comparative Study of Electoral Systems Data

Below we provide the results for five different measures of anti-elite attitudes in Chile using data from the Comparative Study of Electoral Systems (CSES) implemented in 2017. That study incorporates different direct measures of anti-elitism, all of them reporting numbers larger than what we found.

We believe this serves as evidence that supports our approach, which was based on giving people two options for the main division in society (left–right or elite–people) rather than directly asking about anti-elite orientations. We believe using a direct question might overestimate anti-elite attitudes since it is easy for respondents to report such beliefs when evaluating them in isolation.

Table L1: CSES Descriptive Statistics

Question	% Strongly Agree	% Somewhat Agree
Most politicians do not care about people	30.33	44.80
Most politicians are trustworthy	2.2	5.15
Politicians are the main problem of the country	21.10	36.10
People should make the most important policy decisions	22.75	38.25
Most politicians only represent the interests of the rich and powerful	28.55	41.90

14 Appendix N: Days Until the Election

In this appendix, we address a plausible impact of days until the election on anti-elite attitudes. First, we present a descriptive figure, plotting the measures of anti-elite attitudes over days until the election (L3). We do not observe a clear pattern of increasing anti-elite attitudes just before the election in any of the indicators.

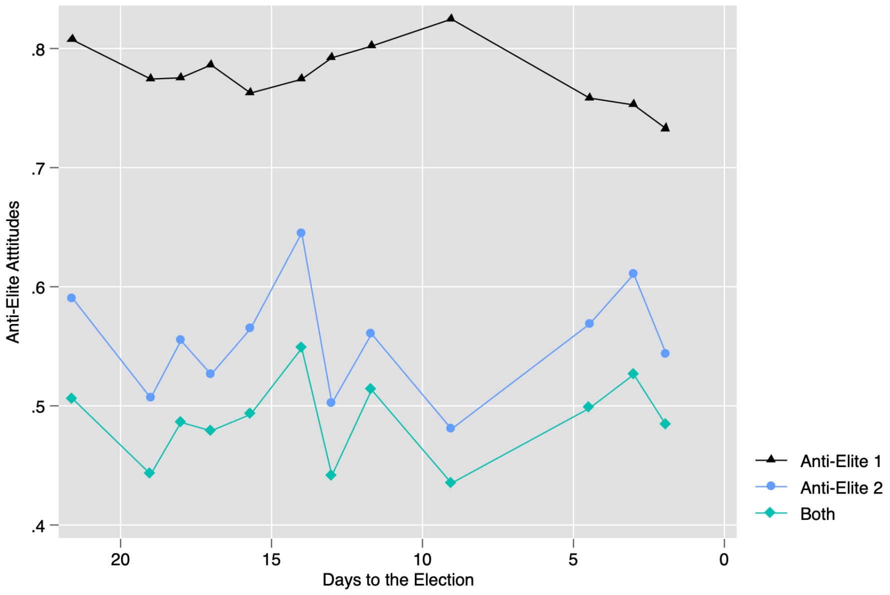

Figure L3: Anti-Elite Attitudes by Days Until the Election
Anti-Elite 1 refers to the question about whether legislators should follow the will of the people, whereas Anti-Elite 2 refers to the question about the main division in society. Both refer to respondents who have anti-elite attitudes in both variables.

In Figure L4, we show the impact of anti-elite attitudes on the preference for independents after controlling for the number of days before the election, and the conclusions remain the same.

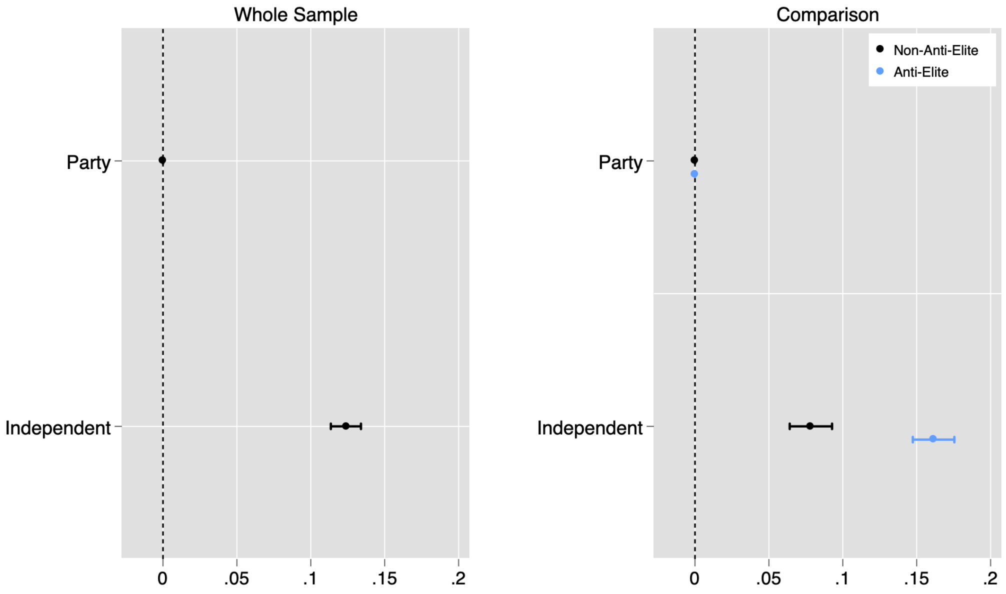

Figure L4: Preference for Independents by Anti-Elite Beliefs Controlling for Days Before the Election

The outcome is the preference for a given candidate. The other conjoint attributes are omitted (see Appendix C for the complete interacted and non-interacted results). The dots represent the point estimates, and the lines 95% confidence intervals. Standard errors are clustered at the respondent level. Number of observations: 39,650 (3,965 survey participants).

References

Altman, D. (2004). Redrawing the chilean electoral map: The presence of socioeconomic and gender factors in the ballot box. *Revista de Ciencia Politica 24*(2), 49.

Araujo, K. (2019). La percepción de las desigualdades: interacciones sociales y procesos socio-históricos. el caso de chile. *Desacatos* (59), 16–31.

Brancati, D. (2008). Winning alone: The electoral fate of independent candidates worldwide. *The Journal of Politics 70*(3), 648–662.

Carreras, M. (2012). The rise of outsiders in latin america, 1980–2010 an institutionalist perspective. *Comparative Political Studies 45*(12), 1451–1482.

Jiménez-Yañez, C. (2020). # chiledespertó: causas del estallido social en chile. *Revista mexicana de sociología 82*(4), 949–957.

Linz, J. J. (1994). *Presidential or parliamentary democracy: does it make a difference?* The Johns Hopkins University Press.

López, M. Á. (2004). Conducta electoral y estratos económicos: el voto de los sectores populares en chile. *Política 43*, 285–298.

Morales, M. (2010). ¿ quiénes son, dónde están, qué quieren?: las bases electorales de los candidatos presidenciales 2009. *Chile 2009: percepciones y actitudes sociales*.

Rovira, C. and R. Castiglioni (2016). Introduction: challenges to political representation in contemporary chile. *Journal of Politics in Latin America 8*(3), 3–24.

Rozas Bugueño, J., A. Olivares, and A. Maillet (2022). Entre la independencia y la militancia: análisis de la vinculación partidaria de los convencionales constituyentes de chile. *Economía y Política 9*(2), 67–100.

Visconti, G. (2021). Reevaluating the role of ideology in chile. *Latin American Politics and Society 63*(2), 1–25.
