# Supplementary material for: Anti-elite attitudes and support for independent candidates
Source: PLoS One. 2023 Oct 12;18(10):e0292098. doi: 10.1371/journal.pone.0292098 (PMC10569503; doi:10.1371/journal.pone.0292098)
Supplement: Attachment — Submitted filename: memo_PONE.pdf [file pone.0292098.s002.pdf]

Response Memo: Anti-Elite Attitudes and Support for Independent Candidates PONE-D-23-15838

Reply to Editor

Thank you very much for the opportunity to revise and resubmit our article to PLOS ONE. We appreciate the reviewers' useful and thoughtful suggestions. We have attempted to address all the comments, which we believe have greatly improved our manuscript.

In this memo, we detail the specific responses to each reviewer point by point and explain how we have addressed their suggestions in the revised manuscript or appendix. Please let us know if there are any further concerns or questions.

Reply to Reviewer 1

1. *I read the paper with great interest. I support the paper moving toward publication.*

   We appreciate the positive remarks about our paper. Thank you.

2. *I think the connection between the election independent candidates and democratic accountability, responsiveness, and executive relations need to be better explained. There is nothing inherently bad about independent politicians, and there is little reason why their election should be a major normative concern. I think maybe the issue is that some independent candidates are anti-system candidates, and that is what the analysis is picking up on.*

   We agree that there is nothing inherently bad about independent politicians, and a more nuanced discussion of the consequences of electing this type of leader is needed.

In the new version of the conclusion, we present some of the positive and negative consequences of electing independent presidents identified in the literature. We summarize them without providing a normative statement about electing this type of political leader. We use these consequences to stress how important it is to understand why people vote for independents since they face different challenges than traditional political leaders. Thanks for the suggestion.

3. *The words "anti-establishment" and "anti-elite" are used interchangeably. Authors should pick their terms, define them, and stick with them.*

   We agree with the reviewer. Now we only use the concept "anti-elite" throughout the manuscript. We define this concept at the beginning of the paper by stating that anti-elite attitudes are "those promoting a moral conflict between the people and the corrupt elite." Using anti-elite attitudes is the most accurate concept since our measurement strategy directly asks about attitudes toward elites. Thank you for this suggestion.

4. *I like the items for capturing anti elite attitudes.*

   Thank you.

5. *More should be done with the text analysis in the main body of the paper.*

   We appreciate this point. We have added a new section in the paper called *Text Analysis,* where we analyze open-ended questions with text data. We asked respondents to define key terms such as "Party Members" and "Independent Politicians." Their answers indicated clear negative attitudes toward elites; they used words such as "thieves" and "corrupt." They characterized independents using descriptions such as "hope" or "change," suggesting that such candidates symbolize values opposite to the political establishment. Thanks for all the comments and suggestions.

Reply to Reviewer 2

1. *I would like to congratulate the authors on this well-written manuscript that investigates the effect of anti-establishment attitudes on the support for independent candidates in Chile using a conjoint experiment. The objective of the study is straightforward, and the manuscript is concise and focused. Overall, I enjoyed reading this paper and I recommend its publication. However, I would like to raise some points that should be addressed.*

   Thank you for the positive and encouraging comments about our manuscript.

2. *Firstly, I suggest being more cautious about whether the measure captures anti-establishment "attitudes". The measure relies on a trade-off question that may be easily influenced by what is currently on top of respondents' mind. They are asked to indicate which of the following is closer to their ideas: 1) the main division in society is between the people and the elite or 2) the main division in society is between the left and the right. If anti-establishment attitudes are more salient, they will pick the former, if left-right conflict is more salient, then they will pick the latter even if their actual attitudes do not change over time. So, it seems like this measure may capture something quite volatile rather than relatively stable anti-establishment attitudes. Authors check whether days to election influences the support for independent candidates. However, I think it would be beneficial to check whether anti-establishment attitudes vary over time and whether, once days to election accounted for, the support for independent candidates among anti-elite and non-anti-elite participants is influenced. Additionally, it would be helpful to examine the correlations between all measures of anti-establishment used in the paper.*

   We agree with the reviewer that these additional robustness checks will benefit the paper. In Appendix N, we have included the descriptive statistics of anti-establishment attitudes over time (days before the election). Importantly, we do not observe a clear pattern of increasing anti-elite attitudes just before the election in any of the indicators. In this appendix we also

included the AMCE regression estimates after controlling for the number of days before the election, and the conclusions remain the same. Finally, in Appendix E we present and discuss the correlations between the two main indicators of anti-establishment attitudes as suggested by the Reviewer.

3. *Secondly, regarding the design, I understand that the initial idea was to prime anti-establishment attitudes using three different treatments (crisis of representation, electoral malfeasance, inequality). The explanation for deviating from the from pre-analysis plan is logical, but I am a little confused about the new design. Were anti-establishment attitudes measured before or after respondents were experimentally primed? If measured before experimental priming, then respondents already thought about whether the division of left versus right or of the people versus the elite is more salient/important before experimental priming. This could also explain the null results reported in Appendix B, as the control group is also primed before answering this question. If measured after experimental priming, then it is problematic, as the main independent variable would be influenced by the design, which would probably lead to an overestimation of the observed effect. Authors should clarify these.*

   Anti-elite attitudes were measured before the prime and conjoint experiment, so this is a pre-treatment measure of anti-elite orientations. We now make this clear in the new version of the manuscript.

   Regarding whether this might have affected the results of the priming/conjoint by priming the control group, we believe this is possible but unlikely. We included a battery of political questions, such as what are the most important attributes of a politician, what should be the next government priorities, and evaluations of multiple political figures, parties, and organizations. As a result, it would be hard for survey respondents to connect one of the multiple pre-treatment questions with the experiment.

   Finally, recent studies have evaluated the existence of “experimenter demand effects” in survey experiments – when participants can infer what the experiment is about and change

their behavior accordingly. Mummolo and Peterson (2019) randomly assign information about the experimenter's intent to survey participants and find that this information does not influence the treatment effects. Thus, even when respondents are able to identify the purpose of the survey experiment, this information does not seem to alter their decisions.

4. *More than 50 percent report anti-establishment attitudes in the sample. Isn't that a little too high? How does it compare to other measures? It would be valuable to discuss whether this may be influenced by the way it is measured and how it compares to findings in the existing literature. Providing context in this regard would be beneficial.*

   We use answers to the anti-elite questions implemented in 2017 by the Comparative Study of Electoral Systems (CSES) to answer the Reviewer's comment. For example, 75% of respondents agreed or somewhat agreed with the statements "Some politicians do not care about the people" and "most politicians care only about the interests of the rich and powerful." The CSES incorporates multiple direct measures of anti-elitism, all of which report larger numbers than what we found (more details in Appendix M).

   We believe this serves as evidence that supports our approach, which was based on giving people two options for the main division in society (left–right or elite–people) rather than directly asking about anti-elite orientations. We believe using a direct question might overestimate anti-elite attitudes since it is easy for respondents to report such beliefs when evaluating them in isolation.

   Finally, we acknowledge that measuring anti-elite attitudes is challenging. Our strategy is based on the Agnes Akkerman index, which involves comparing different options rather than direct measures, to avoid overestimating anti-elite attitudes. Additionally, we use three different approaches. Since our results are robust across all of these measurement strategies, we are confident that the findings are not a consequence of how we capture anti-elite orientations.

5. *Lastly, I would acknowledge the limitations of external validity in the findings (hypothetical*

candidates). In the sample, everyone is more likely to vote for an independent candidate than for a partisan candidate (even those without anti-elite beliefs). It is worth elaborating on how the results generalize to the real context and what patterns we would expect to observe in real world? And do we see those?

Thank you for bringing this to our attention. In the new version, we acknowledge limitations in external validity since people are evaluating hypothetical candidates. However, previous research has compared the findings from conjoint experiments with behavioral benchmarks, showing that both analyses generated similar results. These studies were conducted in different contexts, such as Switzerland (Hainmueller et al., 2015) and Chile (Visconti, 2022).

We would expect to see an increase in the success of independent candidates as anti-elite attitudes are prevalent in Chile. This has been confirmed by some electoral results from previous years, such as the emergence of the "La Lista del Pueblo," and anti-elite/anti-party coalition in the 2020 constitutional assembly elections and the relative success of independent presidential candidates such as Marco Enriquez-Ominami in 2009, Franco Parisi in 2013, and José Antonio Kast in 2017.

In terms of plausible differences between our sample and the population, in Appendix K, we present our main results adjusted by census weights, in order to assign higher weights to observations that are underrepresented in our sample. Thanks for all the feedback.

References

Hainmueller, J., D. Hangartner, and T. Yamamoto (2015). Validating vignette and conjoint survey experiments against real-world behavior. *Proceedings of the National Academy of Sciences 112*(8), 2395–2400.

Mummolo, J. and E. Peterson (2019). Demand effects in survey experiments: An empirical assessment. *American Political Science Review 113*(2), 517–529.

Visconti, G. (2022). After the flood: Disasters, ideological voting and electoral choices in chile. *Political Behavior*, 1–20.
