# Supplementary material for: Anti-elite attitudes and support for independent candidates
Source: PLoS One. 2023 Oct 12;18(10):e0292098. doi: 10.1371/journal.pone.0292098 (PMC10569503; doi:10.1371/journal.pone.0292098)
Supplement: Attachment — Submitted filename: memo_2round.pdf [file pone.0292098.s003.pdf]

Response Memo: Anti-Elite Attitudes and Support for Independent Candidates PONE-D-23-15838R1

Reply to Reviewer 1

1. *In the abstract, the authors claim that anti-elite sentiments have increased. I don't know if there is data showing that. If they don't have it, then cut that claim from the abstract*

   Thanks for this suggestion. In the new version of the manuscript, instead of stating an increase in anti-elite attitudes, we claim that such orientations are prominent in Chile.

2. *Final sentence of the abstract is a bit bland. Any candidate, independent or not, can have "diverse consequences" a range of things. What is it uniquely about independent candidates that is of concern or interest?*

   We agreed with the reviewer. We have included a new sentence with a more precise statement about the consequences of electing independent candidates. Thank you.

Reply to Reviewer 2

1. *The discussion on external validity is essential but comes out of nowhere. I think it will be better as a footnote.*

   Thanks for your comment. Although we agree with your point about the footnote, the journal guidelines impede doing so. Thus, we kept the sentence in the main body, although we improved the transition with the previous paragraph.

2. *The text analysis part is interesting but somehow disconnected. As it is now, authors just describe the table without linking it to what precedes it. Can the authors say something in*

how this complements the main findings and what to take away from these most used words in a sentence or two?

Thanks for your comment. In the new version of the manuscript, we state how this extra analysis complements our main findings. Essentially, including terms people associate with independent candidates allows us to see the meaning of the independent versus party member cues. Thank you!
